# The role of lactate metabolism-related LncRNAs in the prognosis, mutation, and tumor microenvironment of papillary thyroid cancer

**DOI:** 10.3389/fendo.2023.1062317

**Published:** 2023-03-21

**Authors:** Minqi Xia, Shuo Wang, Li Wang, Yingna Mei, Yi Tu, Ling Gao

**Affiliations:** ^1^ Department of Endocrinology & Metabolism, Renmin Hospital of Wuhan University, Wuhan, China; ^2^ Department of Infection Prevention and Control Office, Renmin Hospital of Wuhan University, Wuhan, China; ^3^ Department of Breast & Thyroid Surgery, Renmin Hospital of Wuhan University, Wuhan, China

**Keywords:** papillary thyroid cancer, long non-coding RNAs, lactate metabolism, risk model, immune microenvironment

## Abstract

**Background:**

Lactate, a byproduct of glucose metabolism, is primarily utilized for gluconeogenesis and numerous cellular and organismal life processes. Interestingly, many studies have demonstrated a correlation between lactate metabolism and tumor development. However, the relationship between long non-coding RNAs (lncRNAs) and lactate metabolism in papillary thyroid cancer (PTC) remains to be explored.

**Methods:**

Lactate metabolism-related lncRNAs (LRLs) were obtained by differential expression and correlation analyses, and the risk model was further constructed by least absolute shrinkage and selection operator analysis (Lasso) and Cox analysis. Clinical, immune, tumor mutation, and enrichment analyses were performed based on the risk model. The expression level of six LRLs was tested using RT-PCR.

**Results:**

This study found several lncRNAs linked to lactate metabolism in both The Cancer Genome Atlas (TCGA) and Genotype-Tissue Expression (GTEx) datasets. Using Cox regression analysis, 303 lactate LRLs were found to be substantially associated with prognosis. Lasso was done on the TCGA cohort. Six LRLs were identified as independent predictive indicators for the development of a PTC prognostic risk model. The cohort was separated into two groups based on the median risk score (0.39717 -0.39771). Subsequently, Kaplan-Meier survival analysis and multivariate Cox regression analysis revealed that the high-risk group had a lower survival probability and that the risk score was an independent predictive factor of prognosis. In addition, a nomogram that can easily predict the 1-, 3-, and 5-year survival rates of PTC patients was established. Furthermore, the association between PTC prognostic factors and tumor microenvironment (TME), immune escape, as well as tumor somatic mutation status was investigated in high- and low-risk groups. Lastly, gene expression analysis was used to confirm the differential expression levels of the six LRLs.

**Conclusion:**

In conclusion, we have constructed a prognostic model that can predict the prognosis, mutation status, and TME of PTC patients. The model may have great clinical significance in the comprehensive evaluation of PTC patients.

## Introduction

Thyroid carcer is the most prevalent endocrine tumor in US (1). It has an increasing incidence at an average rate of 4.5% per year (2), which is faster than any other type of cancer. There are four subtypes of thyroid cancer: papillary thyroid cancer (PTC), follicular thyroid cancer (FTC), medullary thyroid cancer (MTC), and anaplastic thyroid cancer (ATC) (3). Among these, the PTC accounts for the majority of the growing incidence, which has more than tripled over the previous four decades (4, 5). Risk stratification is an important component of the PTC treatment strategy, but so far only age at diagnosis is considered the most important risk factor (6). The widespread use of thyroid ultrasound in physical examination has led to the increased detection rate of thyroid cancer annually (7). Fine needle aspiration (FNA) is currently the most effective method of identifying PTC, and it has been used extensively in clinical practice (8). Unfortunately, although FNA generally proves to be effective, there are still many missed diagnoses. In addition, while there have been some advances in diagnostics and treatment, prompt diagnosis and predict prognosis of patient remain inadequate. Therefore, molecular methods are crucial for the early diagnosis of PTC.

Metabolic reprogramming can be caused by mutations in critical metabolic enzymes and is one of the hallmarks of cancer. Targeting cellular metabolism is a potential cancer therapy strategy (9, 10). The Warburg effect refers to the tendency of cancer cells to change their gluconeogenic mode from oxidative phosphorylation to glycolysis and to produce lactate under hypoxic conditions (11). The Warburg effect shields tumor cells from hypoxic conditions and provides a plethora of precursors for the production of nucleic acids (12), fatty acids, and phospholipids that sustain tumor cell proliferation (13).

LncRNAs are non-coding RNAs that exceed 200 nucleotides in length. Recent studies have demonstrated that lncRNAs play a crucial role in carcinogenesis and metastasis, and abnormal expression of lncRNAs have been identified in a range of malignancies. These dysregulated lncRNAs are regarded to be potential biomarkers or therapeutic targets (14–16). However, the role of lactate metabolism -related lncRNAs (LRLs) in PTC development remains to be discovered. In this study, for the first time, a risk model containing six LRLs was constructed and verified with an area under the curve (AUC) value of 0.852-0.979. Moreover, the lncRNA-based risk model was tested with other malignant characteristics such as immune infiltration and tumor somatic mutation. Overall, our work shows that the six-LRLs risk model may serve as a useful prognostic tool for the assessment of PTC patients, and the identified six LRLs can be further studied for possible targeted therapy applications.

## Methods

### Ethical declaration

All subject information was collected from the public database and informed consent was gained by default. This study was approved (WDRY2022-K037) by the Ethics Committee of the Renmin Hospital of Wuhan University, China. Ten PTC tissues and paired paracancer normal tissues were used for *in vitro* validation. Each patient signed an informed consent form.

### Datasets and preprocessing of data

The RNA expression data in counts format, accompanying clinical information, and mutation data of PTC patients (511 cancer and 59 normal) were collected from The Cancer Genome Atlas (TCGA) database (https://portal.gdc.cancer.gov/) (17). Gene expression data of normal thyroid tissues (279 normal tissues) were obtained from the Genotype-Tissue Expression (GTEx) database (https://gtexportal.org/home/) (18). The clinical information of 503 PTC patients is summarized in **Supplementary Table 1**. A total of 260 genes that associated with lactate metabolism and enrichment analysis data set was obtained from MsigDB (http://www.gsea-msigdb.org/gsea/downloads.jsp) (19). Moreover, 300 chemokines genes and 149 immune checkpoint genes were obtained from the NCBI database (https://www.ncbi.nlm.nih.gov/) (20).

### Extraction of LRLs

We used the R package limma (21) and the normalizeBetweenArrays function to reduce the batch effects that may exist between or within the two cohorts and merge the TCGA and GTEx RNA expression data (22). Using the limma package, we evaluated the differential expression of 205 lactate metabolism-related genes (LRGs) in PTC vs. normal patients (|Log_2_FC|>1, FDR<0.05). The 205 LRGs that were differentially expressed in the TCGA-THCA (23) cohort and the 16,877 lncRNAs were then analyzed for relationships using Pearson correlation analysis (P<0.05, correlation coefficient>0.4), from which 4,032 LRLs were identified. Subsequently, the limma package was also used to evaluate the differential expression of LRLs in PTC vs. normal patients (|Log_2_FC|>1, FDR<0.05). Following this, 1,118 differentially expressed LRLs were screened for additional bioinformatic analyses.

### Clustering analysis of LRLs

The ConsensusClusterPlus (24) package in R was used to cluster the data based on the expression of 1,118 LRLs in TCGA-THCA cohort, using a consensus matrix and a cumulative distribution function (CDF) to calculate the optimal number of clusters. The survival analyses on the three clusters were performed using the “survival” package.

### Development of a risk prognosis model

Prognostic LRLs were screened in the TCGA-THCA cohort using univariate Cox regression analysis (P<0.05). To avoid overfitting, a least absolute shrinkage and selection operator (Lasso) regression analysis using the glmnet package (25) was performed. The risk score was calculated using the following formula:


$$\text{Risk Score=}\sum_{\text{i}=1}^{n}\text{coef(i) }*x*(i);$$

where Coef(i) and x(i) represent the respective LRLs coefficient and expression level.

The aforementioned algorithm was used to calculate the risk score for each PTC patient. The characteristics of the risk model were assessed through AUC evaluation and Kaplan-Meier (KM) survival analysis. A risk score was calculated for each patient and the median risk score for the TCGA-THCA cohort was used to define the high- and low-risk groups. The R package scatterplot3d (26) was used to perform principal component analysis (PCA) and construct the relevant plots. The correlation analyses between clinical traits were performed using the survival (27), survminer (28), timeROC (29), and ggplot2 (30) packages in R, combined with the Cox algorithm, KM survival analysis, univariate Cox analysis, multivariate Cox analysis, and ROC curve. Finally, a nomogram was constructed based on the coefficients of the multivariate Cox regression using the rms (31) package.

### Investigating immune infiltration

Using seven different algorithms (XCELL (32), TIMER (33), QUANTISEQ (34), MCPCOUNTER (35), EPIC, CIBERSORT-ABS, and CIBERSORT (36)), the relationship between risk scores and immune cells was estimated. Using single sample gene set enrichment analysis (ssGSEA) and the estimate algorithm, we further compared the immune function and immunological pathways between the high- and low-risk groups. Using the CIBERSORT algorithm, we evaluated the cellular composition of each sample from the high- and low-risk groups to determine the differences in immune cell infiltration between these groups. In addition, the immune escape and tumor microenvironment (TME) between the two groups were compared using the ggpubr (37) and limma packages. Finally, the relationship between immune checkpoints and chemokines expression, as well as the association between risk scores and DNA Stemness Scores (DNAss)/RNA Stemness Scores (RNAss) in the two risk groups were explored using the limma package.

### Evaluation of somatic mutations

The TCGA database was used to extract the somatic mutation profiles of 485 TCGA-THCA patients. Using the maftools (38) package, the mutations in both high- and low-risk groups were analyzed. In this study, the tumor mutation burden (TMB) score was computed by dividing the number of mutations by the length of the exon (30Mb). Based on the median TMB score, all samples with somatic mutations were split into two groups: high and low TMB groups. Variance analysis was performed to assess the differences in TMB between the high- and low-risk groups. Using a correlation scatter plot, the association between risk score and TMB was represented. KM analysis was performed to determine survival probability differences between the high and low TMB groups.

### GO, KEGG, and GSEA enrichment analyses

Gene Ontology (GO) (39), Kyoto Encyclopedia of Genes and Genomes (KEGG) (40), and Gene Set Enrichment Analysis (GSEA) (41) analyses were performed on the risk model using the clusterProfile (42) package. A p-value<0.05 and FDR<0.05 were considered statistically significant in the GO and KEGG analyses. In the GSEA, the GSEA software (version 4.2.3) was obtained from the GSEA website. Subsequently, the samples were divided into two groups based on risk score, and a subset of c2.cp.kegg.v7.5.1.symbols.gmt was obtained from MsigDB to assess the relevant pathways and molecular mechanisms. The minimum gene set was adjusted to 15 and the maximum gene set to 500, with resampling of 1000x. A P-value<0.05, and an FDR<0.25 were considered statistically significant.

### Real-time quantitative polymerase chain reaction (RT-PCR)

Ten PTC tissues and paired paracancer normal tissues were acquired from PTC patients undergoing tumor excision. Fresh tumor and non-tumor tissues were flash-frozen in liquid nitrogen. Total RNA was obtained using an extraction kit (Servicebio, China). Complementary DNA (cDNA) was obtained using the Servicebio^®^ RT First Strand cDNA Synthesis Kit. The SYBR Green qPCR Master Mix (Servicebio) was used for RT-PCR. As an internal control, all expression data were standardized to GAPDH using the 2^−ΔΔCt^ approach. All primers used were outsourced from Servicebio Biotechnology Company (Wuhan, China). Primers for these lncRNAs are included in **Supplementary Table 2**.

### Statistical analyses

The R software (v.4.2.0) and GraphPad Prism (9.0.0) software were used to analyze all the data. GSEA software (version 4.2.3) was used for the GSEA enrichment analysis. To analyze the differences between the two groups, the paired sample t-test was used. Statistical significance was evaluated based on p-values less than 0.05.

## Results

### Study design

**Figure 1:**
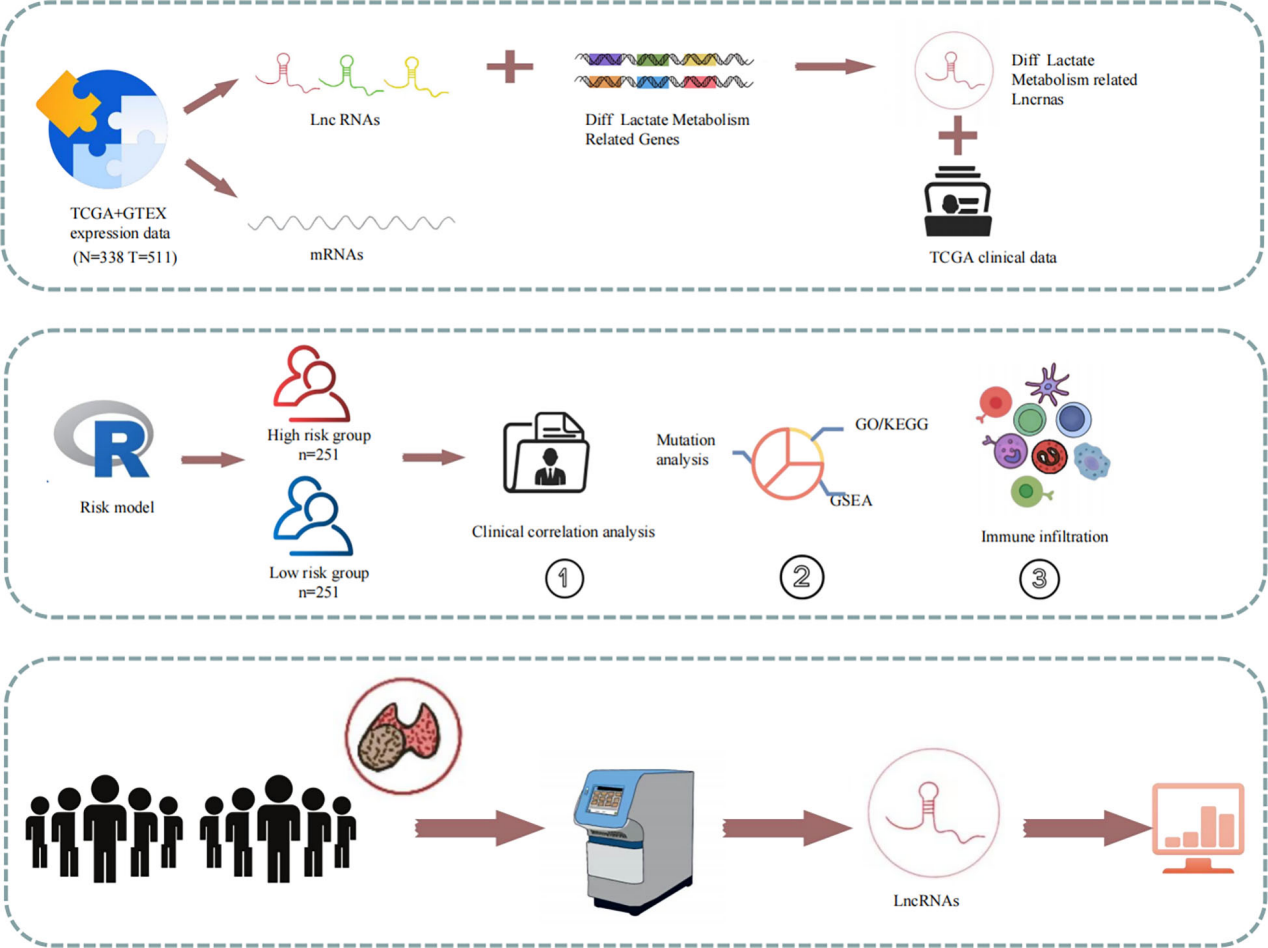
Schematic of the study design. Expression and clinical data from the TCGA database were combined to create a risk model for prognostic, enrichment, mutation, and immune correlation analyses. The expression of the six LRLs was verified through *in vitro* experiments.

The study design is illustrated in **Figure 1**. Using gene expression data from the TCGA and GTEx datasets, 205 differentially expressed LRGs between thyroid normal and PTC tissues were identified. Moreover, 1,118 differentially expressed LRLs obtained from the correlation analysis (P<0.05, correlation coefficient>0.4) and differential analysis (|Log_2_FC|>1, FDR<0.05). Cox regression analysis and Lasso regression analysis were used to obtain the risk prognostic model, which was further used for subsequent clinical, TME, tumor mutation, and enrichment analyses. The expression levels of six LRLs in PTC and normal tissues were verified by RT-PCR.

### LRGs expression levels in the TCGA and GTEx cohorts

**Figure 2:**
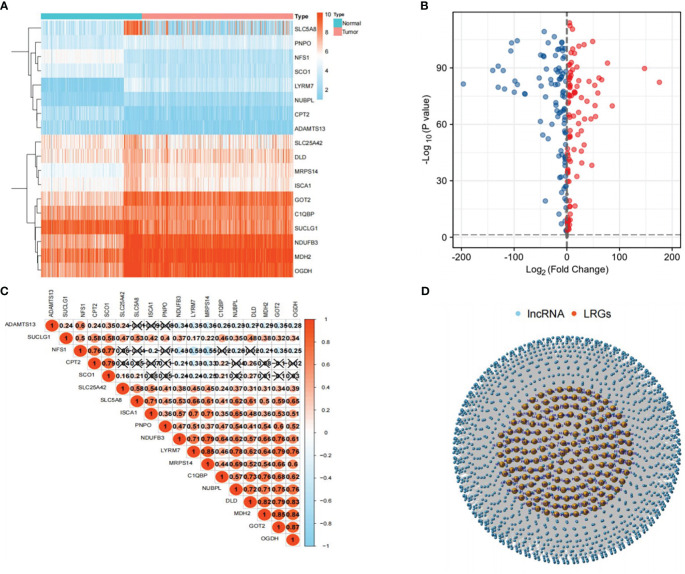
Selection of LRLs. **(A)** Heat map showing the differential expression of 18 genes related to lactate metabolism in tumor and normal groups. **(B)** Distribution of all genes in the tumor and normal groups: the red bubble is log_2_FC>1, and the blue bubble is log_2_FC<-1. The p-value for both red and blue distributed genes was less than 0.001. **(C)** Correlation between 18 LRGs, the darker the color, the stronger the correlation. **(D)** Distribution of LRLs associated with LRGs (cor>0.4, P<0.05).

An evaluation of the expression of 260 LRGs in normal and PTC samples led to the identification of 205 LRGs specific to the TCGA-THCA cohort (|Log_2_FC|>1 and P<0.001). Subsequently, 18 significantly different LRGs were selected to shown in the heatmap (**Figure 2A**). In contrast to normal samples, 104 LRGs were upregulated, and 101 LRGs were downregulated in the PTC samples (**Figure 2B**). To further explore the correlation between LRGs, we performed Pearson correlation analyses. The analyses showed that MDH2 exhibited the largest positive association with GOT2 (r=0.85), while NFS1 had the strongest negative correlation with MRPS14 (r=-0.55) (**Figure 2C**). Using the Pearson correlation analyses, 4,032 LRLs linked with LRGs were identified in the TCGA cohort (correlation coefficient>0.4, P<0.05) (**Figure 2D**).

### Cluster analysis of LRLs

**Figure 3:**
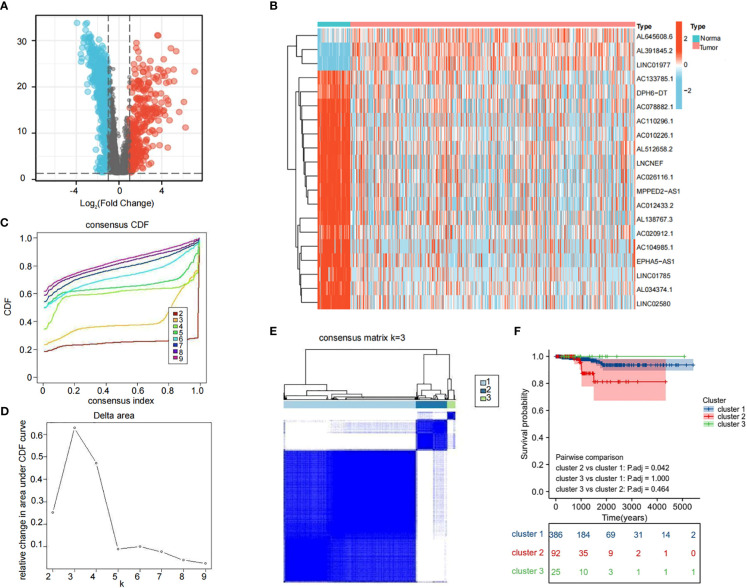
Cluster analysis of LRLs. **(A)** Distribution of LRLs in tumor and normal groups with log_2_FC >1 for the red bubble and log_2_FC<-1 for the blue bubble. The p-value for both red and blue distributions is less than 0.05. **(B)** Differential distribution of 50 differentiated LRLs in normal vs. tumor groups. **(C, D)** Consensus clustering CDF when the data is divided into 2-9 clusters. **(E)** TCGA-THCA cohort RNA expression quantification data were divided into three different clusters. **(F)** KM curves for the three clusters. The OS of cluster 2 was significantly lower than that of cluster 1 (P<0.05). P<0.05 was considered significant.

We obtained 1,118 differentially expressed LRLs (|Log_2_FC|>1, FDR<0.05) in the TCGA-THCA cohort by evaluating the expression levels of 4,032 LRLs in normal and PTC samples using the limma package (**Figure 3A**). The heatmap shows the expression levels of 20 of these LRLs (**Figure 3B**). To further understand the overall role of LRLs in PTC patients, a consensus clustering analysis was performed on the TCGA-THCA cohort. Duplicate samples were removed, and similar samples were grouped into the same category. To determine the optimal number of LRL clusters, we variously tested the total number of LRL clusters from 2 to 9 and examined the CDF curves of the consensus matrix separately. The values with the largest CDF, but with little correlation change were selected (**Figure 3C, D**). Finally, K=3 was used as the optimal number of clusters based on the consensus matrix (**Figure 3E**). KM curves showed a significantly lower survival probability in cluster 2 compared to that of cluster 1 (P<0.05) (**Figure 3F**).

### Evaluation of LRLs and construction of a risk prognostic model

**Figure 4:**
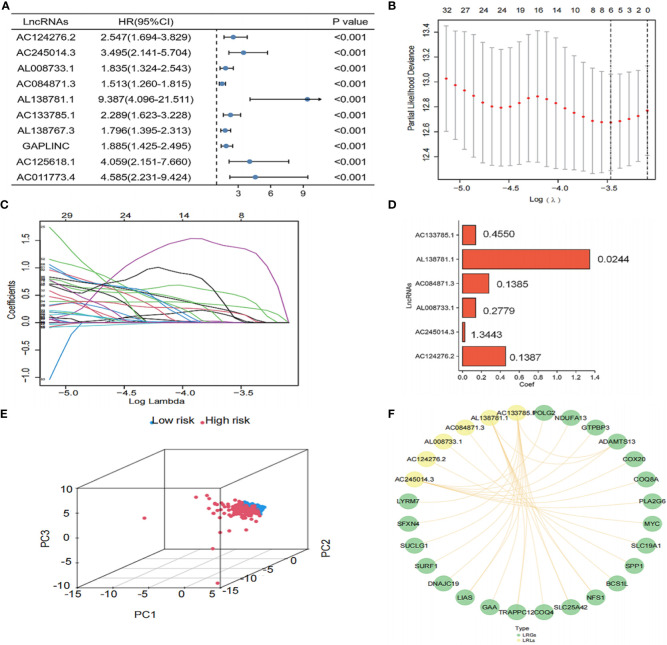
Construction of the risk model. **(A)** Univariate Cox analysis of the differential LRLs. **(B, C)** LRLs with HR<1 and P<0.05 in Cox analysis were selected from the criteria to construct the best predictive signature, and the risk score of the THCA cohort was calculated using the coefficients obtained by the Lasso algorithm. **(D)** Visualization of the coef value. **(E)** A total of 1,118 LRLs in the high- and low-risk groups were identified. **(F)** Visualization of the LRGs and six LRLs of the constructed model (correlation coefficient>0.4, P<0.05). P<0.05 was considered significant.

To further determine the LRLs associated with prognosis among the identified 1,118 differentially expressed LRLs, a univariate Cox analysis was performed. A total of 303 LRLs were screened for further investigation using the limma package (**Figure 4A**). To reduce model overfitting and increase the model’s discriminative ability, the Lasso regression analysis was further applied (**Figure 4B, C**). Subsequently, six LRLs were extracted from the Lasso regression analysis and were used to develop a risk model. The expression and regression coefficients of the six LRLs were then combined to determine the risk scores of the TCGA-THCA patients using the following formula: risk score = (0.455*AC133785.1) + (0.0244*AL138781.1) + (0.1385*AC084871.3) + (0.2779*AL008733.1) + (1.3443*AC245014.3) + (0.1387*AC124276.2) (**Figure 4D**). Based on the median risk score (0.39717-0.39771) of the TCGA-THCA cohort, the patients were separated into the high- and low-risk groups. PCA plots were used to clarify the distribution of LRLs, showing that the median risk score could significantly distinguish the high- and low-risk patients in the TCGA-THCA cohort (**Figure 4E**). The six LRLs that were used to construct the risk model and their related LRGs were then subjected to a Pearson correlation analysis (correlation coefficient>0.4, P<0.05) (**Figure 4F**).

**Figure 5:**
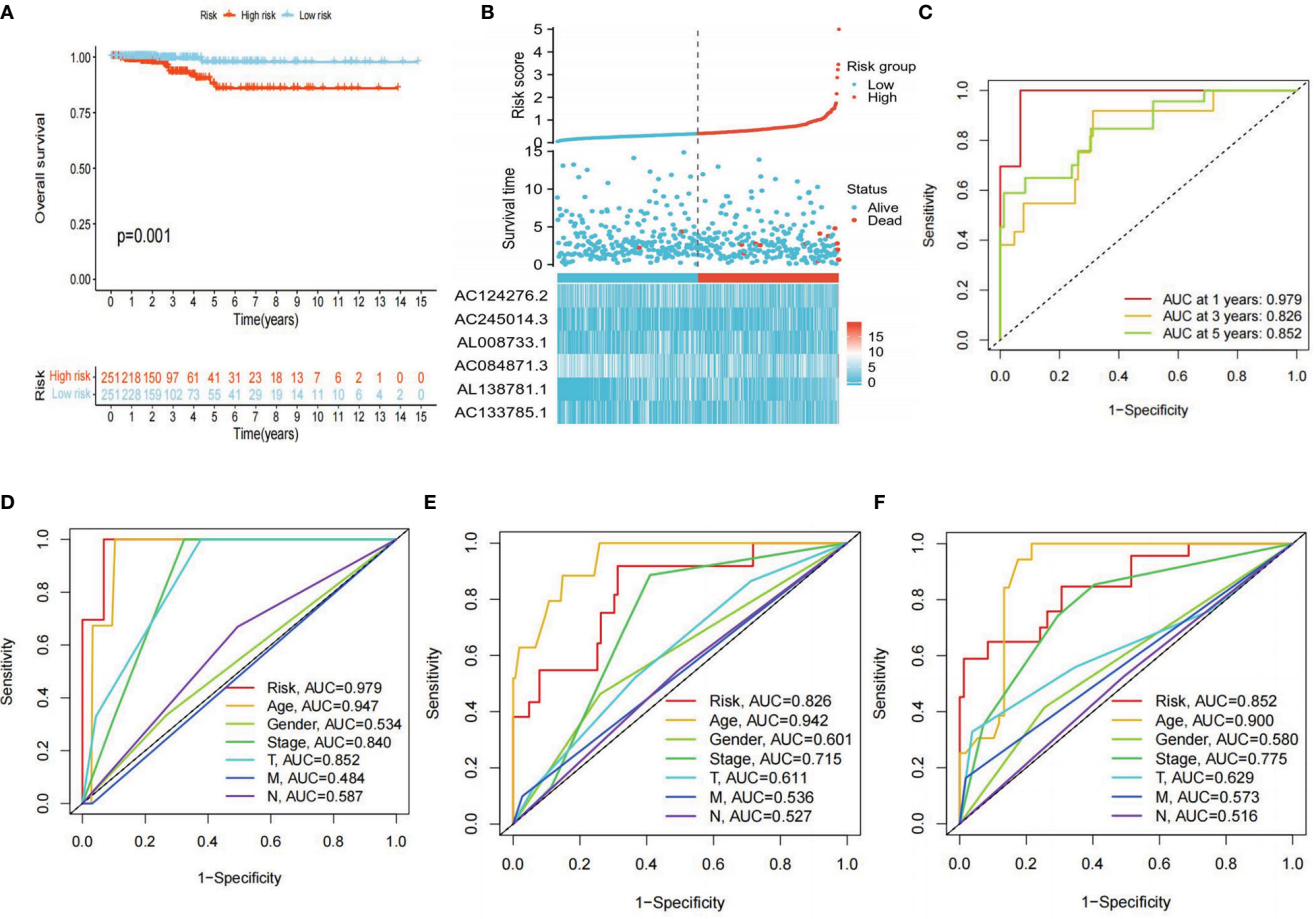
Clinical correlation analysis. **(A)** Differences in OS between high- and low-risk groups (P<0.05 was considered significant). **(B)** Clinical-prognostic-risk factor plots for high- and low-risk groups in the TCGA-THCA cohort. **(C)** AUC values for high- and low-risk groups in the TCGA-THCA cohort at 1, 3, and 5 years, respectively. **(D-F)** The 1, 3, and 5 years ROC curves of each clinical trait in the TCGA cohort.

KM survival analysis showed that the overall survival (OS) of the high-risk group was significantly lower than that of the low-risk group (P<0.01) (**Figure 5A**). Furthermore, the risk scores, survival status, and risk genes expression level were also compared between the high- and low-risk groups in the TCGA-THCA cohort (**Figure 5B**). In addition, the AUC for the risk model were 0.979, 0.826, and 0.852 at 1, 3, and 5 years, respectively (**Figure 5C**). Finally, an examination of the ROC curves showed that risk score had the highest AUC value when compared to other clinical traits, showing the best predictive potential (**Figures 5D–F**).

**Figure 6:**
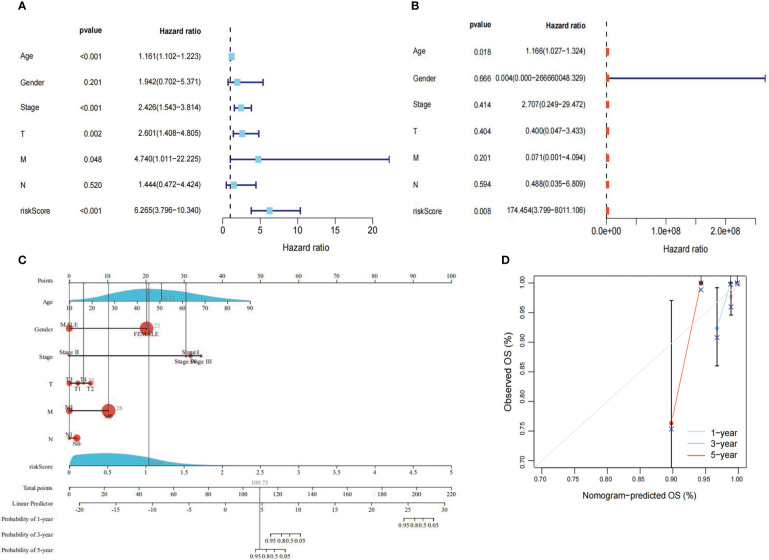
Regression analysis. **(A)** Univariate Cox analysis of the TCGA cohort. **(B)** Multivariate Cox analysis of the TCGA cohort. **(C)** Nomogram of the TCGA cohort. **(D)** Calibration curve of the TCGA cohort nomogram. P<0.05 was considered significant.

To evaluate whether the risk score and clinical traits were independent prognostic factors in PTC patients, univariate and multivariate Cox analyses were performed. The results of the univariate Cox regression analysis showed that the risk score was substantially linked to prognosis in the TCGA cohort (TCGA cohort: HR=6.265, 95% CI=3.796-10.340). Meanwhile, the multivariate Cox regression analysis showed that the risk score remained an independent predictor of prognosis after adjustment for other covariates (TCGA cohort: HR=174.454, 95% CI=3.799-8011.106) (**Figure 6A, B**). In addition, after deleting the missing data in various clinical traits, we integrated data on seven clinical traits, including age, gender, stage, risk score, Tumor, Node and Metastasis (TNM) stage to construct a nomogram to assess the prognostic significance of these traits in 277 PTC patients. The overall C-index of the model was 0.9713, 95% CI (0.9224-1), p-value=1.6427e-79 (**Figure 6C**). Moreover, the prediction curve for PTC patients was close to the standard curve, indicating that the expected survival at 1-, 3-, and 5-years was similar to the actual survival at these time points (**Figure 6D**).

**Figure 7:**
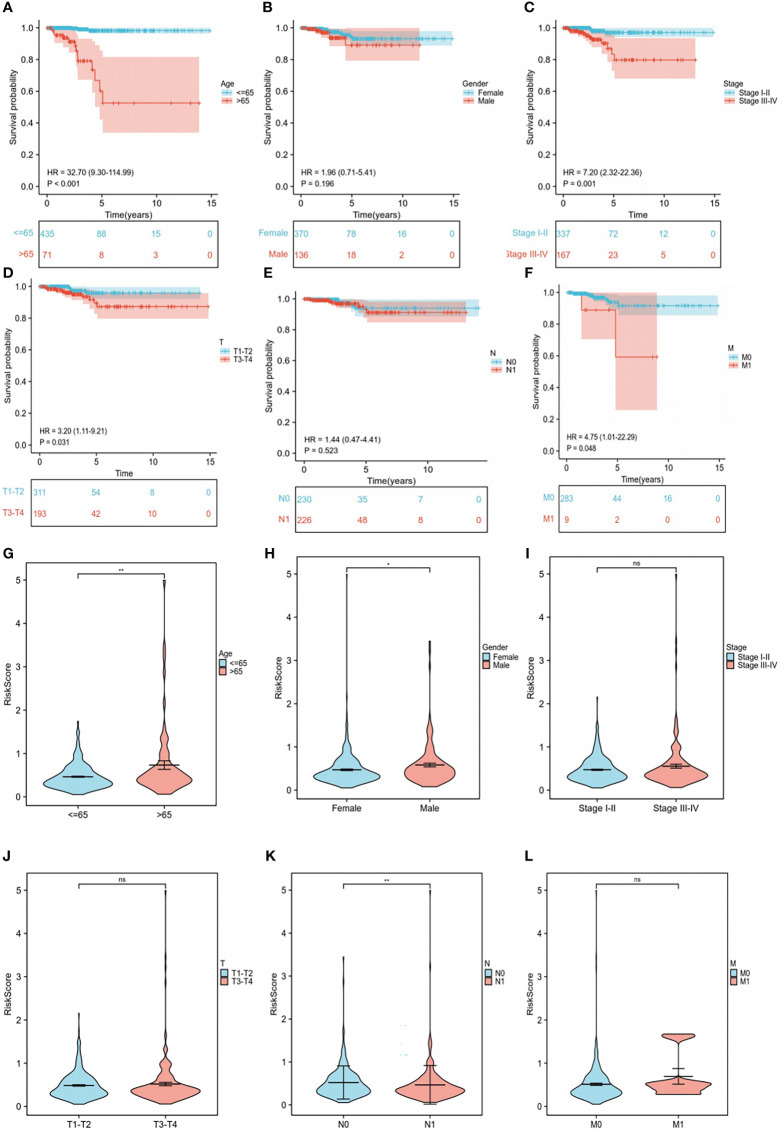
Subgroup clinical trait analysis. **(A-F)** Association of each clinical trait (age, gender, stage, TNM stage) with survival probability. **(G-L)** Association of each clinical trait (age, gender, stage, TNM stage) with risk score. "ns" means “no significance” *P<0.05, **P<0.01, P<0.05 was considered significant.

To further explore the relationship between each clinical trait, survival probability, and risk score, clinical data such as age, gender, stage, and TNM stage were extracted separately. Samples with missing information were removed, and the remaining samples were subjected to Cox regression analysis to determine the correlation of clinical traits with survival probability and risk score, respectively. Our results showed that survival probability was significantly lower in patients >65 years of age compared to those<=65 years (P<0.001) (**Figure 7A**). Moreover, stage I-II, T1-T2, and M0 patients have a significantly higher survival probability than stage III-IV, T3-T4, and M1 patients (P<0.05), respectively. In contrast, gender and N stage did not seem to have a significant effect on survival (**Figures 7B–F**). Furthermore, significant differences in risk scores between the high- and low-risk groups in terms of gender, age, and N stage were observed (P<0.05). More specifically, patients with age >65 years, men, and patients with N1 stage had higher risk scores than patients with age<=65 years, women, and patients with N0 stage, respectively (P<0.05). Finally, stage I and II, T1-T2 stage, and T3-T4 stage did not seem to have a significant effect on risk scores (**Figures 7G–L**).

### Tumor-infiltrating immune cell profiles

**Figure 8:**
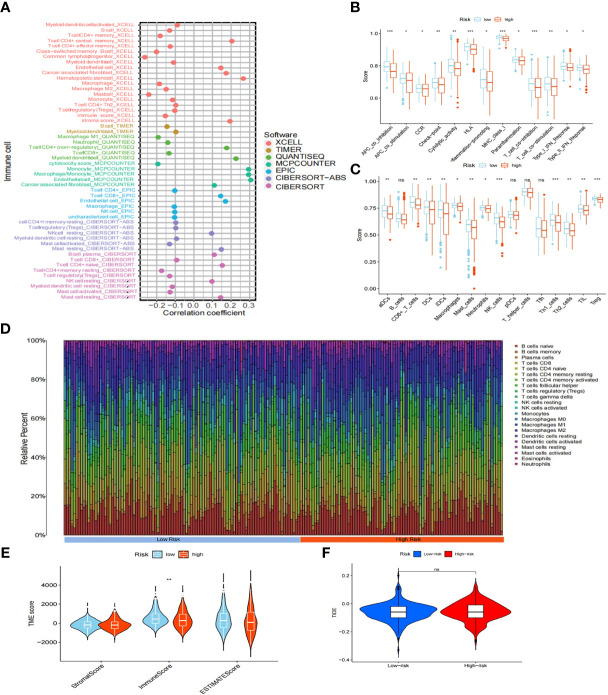
Immuno-infiltration analysis. **(A)** Association between risk scores and immune cells under seven algorithms (XCELL, TIMER, QUANTISEQ, MCPCOUNTER, EPIC, CIBERSORT-ABS, CIBERSORT). **(B)** Correlation between high- and low-risk groups and various immune functions in PTC and normal tissues. **(C)** Correlation between high- and low-risk groups and various immune cells in thyroid tumor and non-tumor tissues. **(D)** Distribution of cells in each sample in the high- and low-risk groups. **(E)** TME score for high- and low-risk groups. **(F)** TIDE score for high- and low-risk groups. "ns" means “no significance” *P<0.05, **P<0.01, ***P<0.001, P<0.05 was considered significant.

We evaluated the relationship between immunity and risk score. First, using seven different algorithms (XCELL, TIMER, QUANTISEQ, MCPCOUNTER, EPIC, CIBERSORT-ABS, CIBERSORT), we compared the correlation between risk score and various immune cells expression level. Our analyses revealed that endothelial cells, cancer-associated fibroblasts, monocytes, and the remaining CD4+ T cells were positively correlated with risk score (**Figure 8A**). The generated box plots show that the high-risk group had an immune cell proportion that was significantly lower than that of the low-risk group when 16 immune cell types and their associated functions were analyzed using the ssGSEA algorithms (**Figure 8B, C**). Next, the various cellular composition of each sample in the high- and low-risk groups were measured using the CIBERSORE algorithm (**Figure 8D**). Our results showed that the immune score was significantly lower in the high-risk group than in the low-risk group (P<0.05) (**Figure 8E**). Finally, the Tumor Immune Dysfunction and Exclusion (TIDE) results showed that there was no significant difference in immune escape between the high- and low-risk groups (**Figure 8F**). Taken together, the results showed that the low-risk group had higher immune cell infiltration.

### Analysis of immune checkpoints and chemokines

**Figure 9:**
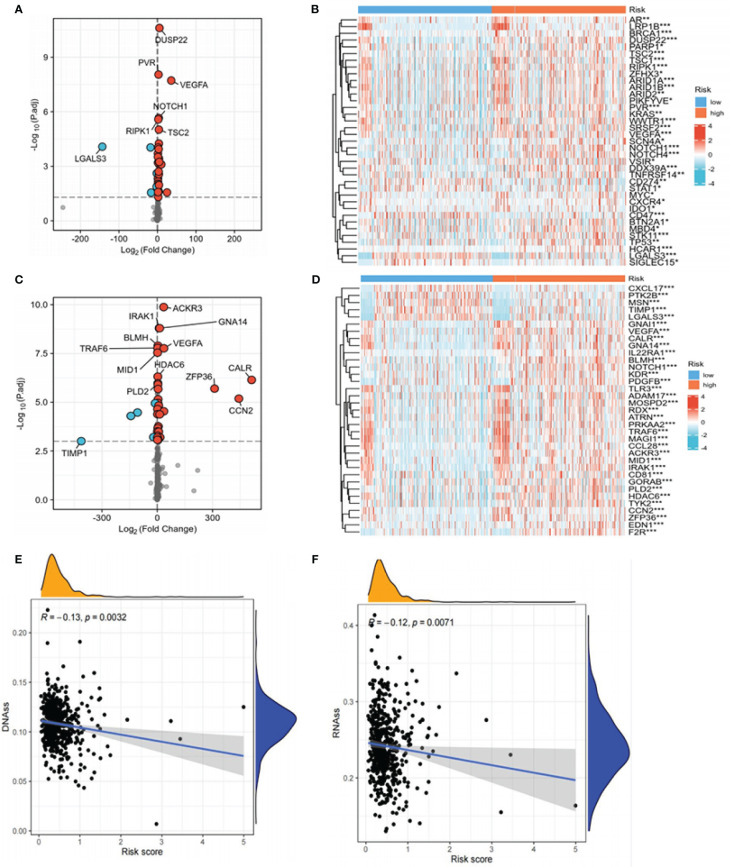
Expression of immune checkpoints, chemokines, DNAss, and RNAss in high- and low-risk groups. **(A)** Volcano plot of the distribution of immune checkpoint-associated genes in high- and low-risk groups. The right side of the vertical axis is P<0.05, log_2_FC >1, while the left side is P<0.05, log_2_FC<-1. **(B)** Heat map of the distribution of immune checkpoint-associated genes in the high- and low-risk groups. **(C)** Volcano plot of the distribution of chemokines in high- and low-risk groups. The right side of the vertical axis is P<0.05, log_2_FC >1, and the left side is P<0.05, log_2_FC<-1. **(D)** Heat map of the distribution of chemokines in the high- and low-risk groups. **(E, F)** The relationship between DNAss, RNAss, and risk scores (*P<0.05, **P<0.01, ***P<0.001, P<0.05 was considered significant).

Considering the relevance of human chemokines and checkpoint immunotherapy, it is also crucial to note that there were considerable differences in the expression of immunological checkpoints and chemokines between the different risk groups (**Figures 9A–D**). Increasing evidence reveals a link between the increased expression of stemness-related markers in tumor cells and drug resistance, cancer recurrence, and tumor development (43). Our results demonstrated an inverse relationship between risk scores and DNAss/RNAss (P<0.01) (**Figures 9E, F**).

### Evaluation of somatic mutations in tumors

**Figure 10:**
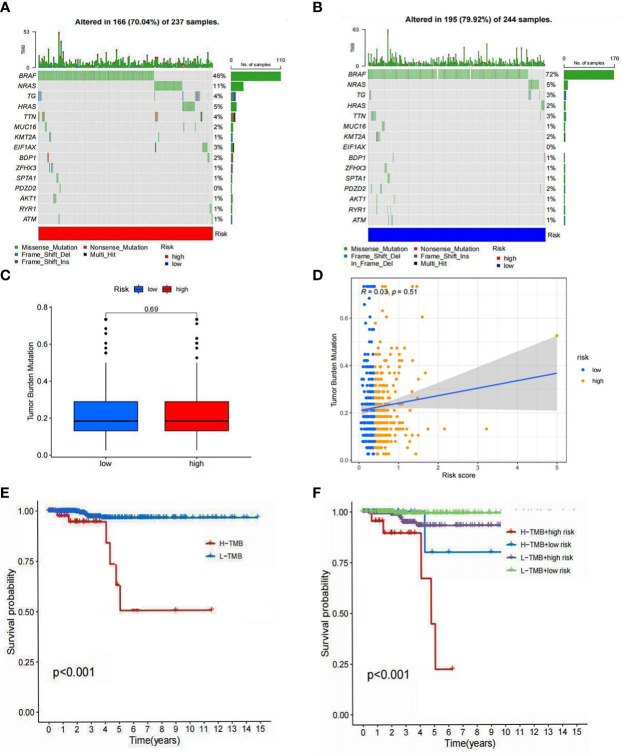
Tumor mutation analysis. **(A)** The 15 mutations were identified in the high-risk group. **(B)** The 15 mutations were identified in the low-risk group. **(C)** Differences in the TMB of high- and low-risk groups. **(D)** Relationship between risk scores and TMB. **(E)** Differences in survival between the high TMB and low TMB groups. **(F)** Differences in survival between high TMB with a low-risk score, high TMB with a high-risk score, low TMB with a low-risk score, and low TMB with a high-risk score (P<0.05 was considered significant).

Since mutations play critical roles in cancer development, we focused on the distribution of somatic mutations in the two risk groups. The 15 most commonly mutated genes in both populations were identified (**Figure 10A, B**). No significant difference in TMB was observed between the high- and low-risk groups, and no strong correlation between TMB and risk score was seen (**Figure 10C, D**). KM analysis showed a better survival probability in the low TMB group than in the high TMB group (P<0.001) (**Figure 10E**). Furthermore, patients with low risk and low TMB scores had the best prognosis (P<0.001) (**Figure 10F**), indicating that survival probability is negatively correlated with risk scores and TMB. Overall, immunotherapy for PTC patients with high TMB appears to be ineffective. This may be related to the limited data on tumor mutations in PTC patients and warrants further studies to explore the relationship between TMB and immunotherapy.

### Enrichment analysis

**Figure 11:**
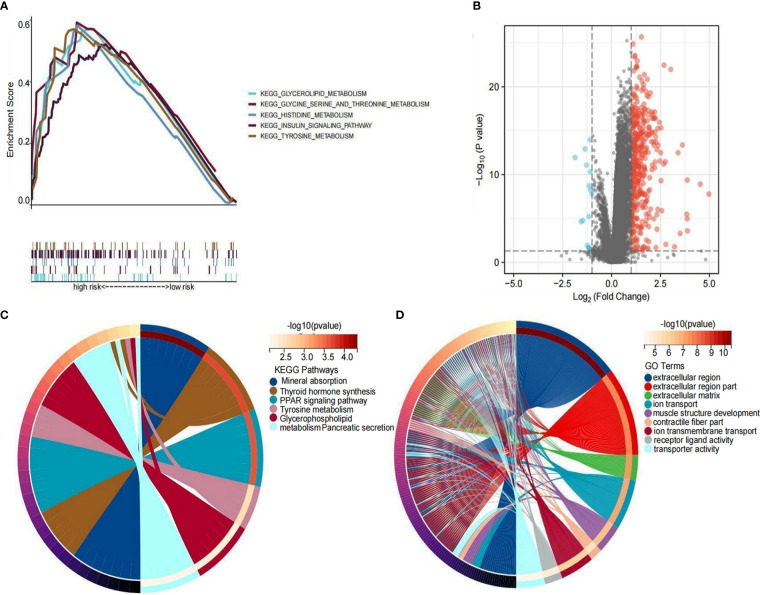
Functional enrichment analysis. **(A)** GSEA on the high-risk group. **(B)** Distribution of differential LRLs between the high- and low-risk groups: log_2_FC >1 for the red bubble and log_2_FC<-1 for the blue bubble. **(C)** KEGG enrichment analysis of the differentially expressed genes between the high- and low-risk groups. **(D)** GO enrichment analysis of the differentially expressed genes between the high- and low-risk groups.

To elucidate the underlying biological functions and pathways associated with the six LRLs, a GSEA enrichment analysis was performed. The results showed that the high-risk group was mainly enriched in amino acid metabolism and insulin metabolism pathway, while the low-risk group was not enriched in these pathways (**Figure 11A**). Based on the different gene expression of high- and low-risk group data (|Log_2_FC|>1 and P<0.05), 418 risk differentially expressed genes (DEGs) were identified (**Figure 11B**). KEGG enrichment analysis showed that risk DEGs were mainly enriched in amino acid metabolism, glycerol phospholipid metabolism, and thyroid hormone synthesis (**Figure 11C**). Moreover, GO enrichment analysis showed that these risk DEGs were mainly related to extracellular matrix and ion transport (**Figure 11D**). Taken together, the six LRLs were found to be mainly related to amino acid metabolism and extracellular matrix.

### Expression analysis of LRLs

**Figure 12:**
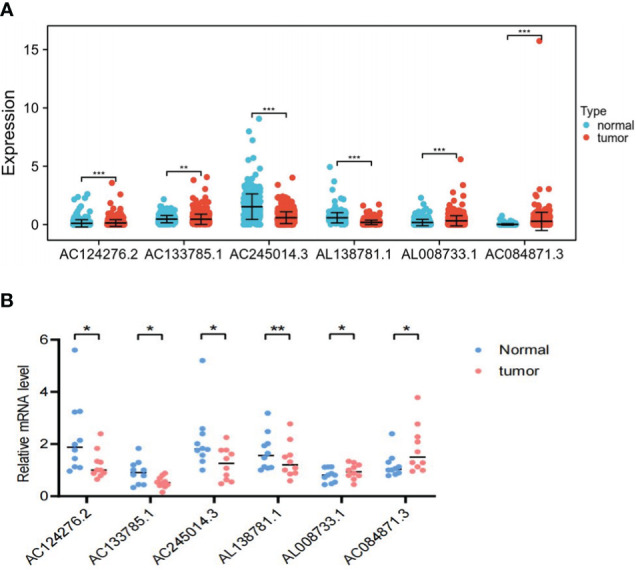
*In vitro* experimental validation. **(A)** Differential expression of the six LRLs (AC124276.2, AC133785.1, AC245014.3, AL138781.1, AL008733.1, and AC084871.3) in the tumor and normal group. **(B)** The RT-PCR results show the expression of the six LRLs in 10 pairs of PTC and normal tissues. *p<0.05, **p<0.01, ***p<0.001, p<0.05 was considered significant.

To further illustrate the viability of our prognostic model, we analyzed the expression levels of the six LRLs in TCGA-THCA cohort. Consistent with the RNA-Seq data, the results showed that AC084871.3 and AL008733.1 were upregulated, while AC133785.1, AL138781.1, AC245014.3, and AC124276.2 were significantly downregulated in PTC samples compared with normal counterparts. These findings collectively strengthen the validity and dependability of the risk model (**Figure 12A, B**).

## Discussion

To our knowledge, this is the first study to investigate the relationship between LRLs and PTC. In this study, we used multiple public databases to explore the predictive potential of novel biomarkers in PTC. A total of 1,118 LRLs were initially identified by differential expression analysis on PTC and normal samples. The top six LRLs (AC084871.3, AC133785.1, AL138781.1, AL008733.1, AC245014.3, and AC124276.2) were identified using Lasso and Cox regression analyses. The risk model for the six-LRLs signature accurately predicted the OS of PTC patients, demonstrating its potential as an independent prognostic model. Meanwhile, the AUC value of the risk model reached 0.979, which was significantly higher than the prognostic model constructed in previous studies (44–46), indicating that our six-LRLs signature is a better prediction model. Based on the risk model, differences in enrichment pathways, immune function, immune escape and mutations were compared between the high- and low-risk groups. Our findings can contribute to the design of small molecule targeting drugs based on the differences in the expression of immune infiltration, immune checkpoints, and chemokines between the two risk groups, with the potential to improve chemotherapy, radiotherapy, and even immunotherapy. Our model also provides a new strategy for risk score-based PTC prognostic assessment.

In this study, we identified three clusters based on the expression of 1,181 LRLs. Cluster 2 had a worser OS compared to cluster 1, and there was not significance difference in the effect on survival probability between the cluster1 and cluster3, cluster2 and cluster3. Unfortunately, the results showed that the cluster analysis did not seem to separate the three clusters, hence further risk modeling was carried out. Our risk model was composed of six LRLs (AC084871.3, AC133785.1, AL138781.1, AL008733.1, AC245014.3, and AC124276.2), and the risk score of each patient was calculated based on the expression level of these LRLs. However, the roles of these LRLs in other diseases or cancer types have not yet been reported. Early studies have shown that an increasing number of LRLs are associated with tumor growth and immune escape. For instance, decreases lactate generation by inhibiting PKM2 oligomerization, hence lowering cancer growth and macrophage polarization (47).The lncNSPL facilitated influenza virus a immunological escape by inhibiting trim25-mediated RIG-I ubiquitination of the k63 linkage (48). Another study has found that during oral carcinogenesis, LPS/TLR4-activated lncRNA IFITM4P can promote immune escape by upregulating PD-L1 through a dual mechanism (49). Therefore, the six LRLs we found are promising biomarkers in the future. Next, our further research showed that risk score was considered as a risk factor for the prognosis of TCGA-THCA patients. By combining our analysis of clinical traits, we also found that age and risk score were independent risk factors for the prognosis of PTC patients (HR>1, P<0.01). Moreover, age and risk scores were significantly associated with survival probability. This is consistent with the findings of previous studies (50).

In addition to examining the impact of risk model on the survival prognosis of PTC patients, we also investigated the effect on the TME of PTC patients. In our research, ssGSEA analysis revealed that 12 types of immune cells were significantly reduced, while 13 types of immune signals were significantly suppressed in the high-risk group. Consistent with the results of our immunological studies, there are considerable disparities in the immune responses between the high- and low-risk groups. One of the most prevalent innate immune cells that act as an antitumor response is the macrophage (51). Macrophages play a crucial part in the progression or resolution of PTC, which is often surrounded by a significant number of immunological “reactive” cells (52–54). Traditionally, macrophage can become M1-phenotype and can enhance both innate and adaptive immunity when activated by a variety of environmental stimuli like bacterial LPS and IFN-γ (51). M2-like macrophages suppress the immune system by producing immunosuppressive molecules such as HLA-G, IL-10, and TGF-β (55). Specifically, the M2/repair macrophages promote tumor development, while the M1/kill macrophages block or delay tumor growth (56). Our study showed that macrophage expression was negatively correlated with risk score, meaning that as risk score increased, macrophage expression gradually decreased, resulting in a significantly lower macrophage immune score in the high-risk group than in the low-risk group. Therefore, we infer that the significantly lower survival probability of patients in the high-risk group compared to those in the low-risk group may be related to the significantly lower expression of M1/kill macrophages. Studies have shown that pro-tumor M2 macrophages can be changed into anti-tumor M1 macrophages using some immunotherapeutic techniques (57, 58). The macrophage immune score in the high-risk group was lower than that in the low-risk group, which may be related to the significantly decreased M1 macrophage expression in the macrophages, resulting in a higher mortality in the high-risk group. Our work may give a fresh perspective on PTC function and immune mechanism processes.

Some of the upregulated immune checkpoints, including CXCR4, are expected to become new immunotherapeutic targets, which can achieve anti-tumor effects together with PD-1/PD-L1 inhibitors. CXCR4 is overexpressed in more than 20 cancer types (59–62). It has been reported that CXCL12 can mobilize cancer cells *via* CXCR4 (63). NF-κB stimulates the expression of the chemokine receptor CXCR4 to encourage the migration and metastasis of breast cancer cells (64). When CXCR4 receptors are present in large numbers on cancer cells, it can ensure the migration of cancer cells, thus laying the foundation for cancer metastasis (65). Tailored drug strategies for cancer patients may prove to be important. These results imply that this prognostic characteristic may aid in elucidating the regulatory mechanisms of tumor immunity and provide novel insights for future TME research.

Tumor mutation analysis showed that gene mutations in the high- and low-risk groups were mainly concentrated in missense mutations of the BRAF gene. Furthermore, there was no significant difference in the TMB score between high- and low-risk groups. TMB values can vary considerably between tumor types as well as within the same tumor type. In the TCGA-THCA cohort, there was little difference in TMB between the high- and low-risk groups, suggesting that there may be insignificant effects of single immunosuppressive therapy on the treatment of the 2 groups. TMB may have its greatest utility when combined with other biomarkers such as PD-L1 and T-cell inflammatory markers.

The GSEA showed that the main functions of the LRLs in the high-risk group were mainly enriched in amino acid metabolism and insulin metabolism. In addition, KEGG analysis revealed that the distinctions between the high- and low-risk groups centered on amino acid metabolism, glycerol phospholipid metabolism, and thyroid hormone synthesis. These were also true for the GO enrichment analysis, with an additional concentration on the extracellular matrix. The amino acid metabolism is distinct since it connects both carbon and nitrogen metabolism, and amino acids are important intermediates in many metabolic processes (66). It is established that most amino acids undergo a series of transformations to produce sugars, which in turn can produce lactic acid under anaerobic conditions. In other words, amino acid metabolism is closely related to lactate metabolism. Meanwhile, the extracellular matrix invasion is crucial in the early stage of the metastatic cascade. Matrix metalloproteinase inhibition can slow the deterioration of the extracellular matrix and prevents the spread of primary tumor cells (67, 68). It has been reported that extracellular matrix signaling protein ADAM22 can facilitate distant disease spread *in vivo* and is a crucial mechanism in the metastasis of breast cancer (69). High expression of ECM-related gene COL6A1 predicts poor prognosis and poor immunotherapy response in bladder cancer (70). Overall, there are altered expression patterns of extracellular matrix-associated genes in metastatic cancers, and extracellular matrix-associated genes are expected to be markers of aggressive, growing tumors.

PTC continues to be treated with surgery as the first-line therapy, but immunotherapy is gaining importance for refractory thyroid cancer (71, 72). In the United States, there were about 62,450 cases of differentiated thyroid cancer diagnosed in 2015 (73). Moreover, it was estimated that about 25-50% of patients with locally advanced or metastatic disease would have a refractory response to radioiodine (74). Although well-differentiated thyroid cancer responds well to radioiodine therapy and often has favorable treatment outcomes, the management of refractory differentiated thyroid cancer remains a major challenge and is a leading cause of death in thyroid cancer patients, with no effective treatment (75, 76). To our knowledge, this is the first study to construct a predictive risk model for PTC patients based on six LRLs. We also systematically evaluated the functional enrichment, immune cell infiltration, and tumor somatic mutations of the model, adding rigor to this study. Despite its numerous benefits, the present study has several drawbacks. First, all data used are from retrospective research data, and additional prospective, large-scale, multicenter trials are required to give more compelling evidence for the therapeutic implications of our study. Moreover, since the LRLs screened in this work have not yet been subjected to relevant carcinogenic mechanisms, additional research is required to determine their regulatory processes.

## Conclusion

The six-LRLs risk model developed in this work is a consistent and strong predictor of PTC patient survival outcomes. This characteristic was also closely associated with TMB and immune checkpoints can be used as a treatment target for future immunotherapy or drug development.

## Data availability statement

The original contributions presented in the study are included in the article/**Supplementary Material**. Further inquiries can be directed to the corresponding authors.

## Ethics statement

The studies involving human participants were reviewed and approved by Ethics Committee of the Renmin Hospital of Wuhan University. Written informed consent to participate in this study was provided by the participants’ legal guardian/next of kin. The animal study was reviewed and approved by Ethics Committee of the Renmin Hospital of Wuhan University.

## Author contributions

MX and YM performed the data analysis and wrote the manuscript. SW,LW and LG participated in the study design,construction of figures, and manuscript writing and revision. YT provided the clinical samples for gene expression analysis and helped to prepare the manuscript. All authors contributed to the article and approved the submitted version.

## Supplementary material

Supplementary Table 1 The baseline information of patients. The clinical information of all TCGA-THCA patients, including survival status, age, gender,stage,T,N,M.

Supplementary Table 2 Primers for lncRNAs. Primer information of AC084871.3, AC133785.1, AL138781.1, AL008733.1, AC245014.3, and AC124276.2.
