# Supplementary material for: The role of lactate metabolism-related LncRNAs in the prognosis, mutation, and tumor microenvironment of papillary thyroid cancer
Source: Front Endocrinol (Lausanne). 2023 Mar 21;14:1062317. doi: 10.3389/fendo.2023.1062317 (PMC10070953; doi:10.3389/fendo.2023.1062317)
Supplement: Supplementary Table 1 — The baseline information of patients. The clinical information of all TCGA-THCA patients, including survival status, age, gender,stage,T,N,M. [file DataSheet_1.pdf]

Characteristic	TCGA-THCA(n=503)	Percentage
**Survival status**		
Alive	487	96.80%
Dead	16	3.20%
**Age**		
<=65	432	85.90%
>65	71	14.10%
**Gender**		
Female	368	73.20%
Male	135	26.80%
**Stage**		
Stage I	283	56.50%
Stage II	52	10.40%
Stage III	111	22.20%
Stage IV	55	11%
**T**		
T1	143	28.50%
T2	166	33.10%
T3	169	33.70%
T4	23	4.60%
**N**		
N0	228	50.30%
N1	225	49.70%
**M**		
M0	282	96.90%
M1	9	3.10%
