# Supplementary material for: The role of lactate metabolism-related LncRNAs in the prognosis, mutation, and tumor microenvironment of papillary thyroid cancer
Source: Front Endocrinol (Lausanne). 2023 Mar 21;14:1062317. doi: 10.3389/fendo.2023.1062317 (PMC10070953; doi:10.3389/fendo.2023.1062317)
Supplement: Supplementary Table 2 — Primers for lncRNAs. Primer information of AC084871.3, AC133785.1, AL138781.1, AL008733.1, AC245014.3, and AC124276.2. [file DataSheet_2.pdf]

| LncRNAs | Forward | Reverse |
|---|---|---|
| AC124276.2 | CCTGCCTTCCTCTTCTCTCT | TTCTTCTTTCTGGTGCTTCC |
| AC245014.3 | GAGGCATATCTGACCCCTACT | ACTGGAAAACATGGCAAAAT |
| AL008733.1 | TAACAGGGAGAGCCATTTGT | CTGTACGCCATCAGGATCTT |
| AC084871.3 | AGGGTCTCACTATGTTGCTCA | CAATCTCTACCTCCTGGGTTC |
| AL138781.1 | TGTCTACCTGGCTCTCTGTG | TCGTAAGGATGATGTATGGAAC |
| AC133785.1 | GTCACGCTCCTCAGTTCTCT | CAAACAAACACAACACACACC |
